# RETRACTION: Application of VSD Technique in Adults With Chronic Osteomyelitis of the Extremities Combined With Soft Tissue Defects

**DOI:** 10.1111/iwj.70673

**Published:** 2025-04-23

## Abstract

**RETRACTION:** ZhangX. , ChenY. , XiaoX. , WangZ. , YangX. , and ZhaoZ. , “Application of VSD Technique in Adults With Chronic Osteomyelitis of the Extremities Combined With Soft Tissue Defects,” International Wound Journal 20, no. 3 (2023): 768–773, 10.1111/iwj.13921.36382601 PMC9927891

The above article, published online on 09 August 2022, in Wiley Online Library (http://onlinelibrary.wiley.com/), has been retracted by agreement between the journal Editor in Chief, Professor Keith Harding; and John Wiley & Sons Ltd. Following an investigation by the publisher, all parties have concluded that this article was accepted solely on the basis of a compromised peer review process. The editors have therefore decided to retract the article. The authors did not respond to our notice regarding the retraction.

**RETRACTION:** X. Zhang , Y. Chen , X. Xiao , Z. Wang , X. Yang , and Z. Zhao , “Application of VSD Technique in Adults With Chronic Osteomyelitis of the Extremities Combined With Soft Tissue Defects,” International Wound Journal 20, no. 3 (2023): 768–773, 10.1111/iwj.13921.36382601 PMC9927891

The above article, published online on 09 August 2022, in Wiley Online Library (http://onlinelibrary.wiley.com/), has been retracted by agreement between the journal Editor in Chief, Professor Keith Harding; and John Wiley & Sons Ltd. Following an investigation by the publisher, all parties have concluded that this article was accepted solely on the basis of a compromised peer review process. The editors have therefore decided to retract the article. The authors did not respond to our notice regarding the retraction.

